# Convergence of pathway analysis and pattern recognition predicts sensitization to latest generation TRAIL therapeutics by IAP antagonism

**DOI:** 10.1038/s41418-020-0512-5

**Published:** 2020-02-21

**Authors:** Vesna Vetma, Cristiano Guttà, Nathalie Peters, Christian Praetorius, Meike Hutt, Oliver Seifert, Friedegund Meier, Roland Kontermann, Dagmar Kulms, Markus Rehm

**Affiliations:** 1 Institute of Cell Biology and Immunology, University of Stuttgart, Stuttgart, Germany; 2 Department of Physiology & Medical Physics, Royal College of Surgeons in Ireland, Dublin, Ireland; 3 Center for Regenerative Therapies, Technical University Dresden, Dresden, Germany; 4 Skin Cancer Center at the University Cancer Centre, Department of Dermatology, Faculty of Medicine, University Hospital Carl Gustav Carus, Technical University Dresden, Dresden, Germany; 5 National Center for Tumor Diseases (NCT), Dresden, Germany; 6 German Cancer Research Center (DKFZ), Heidelberg, Germany; 7 Stuttgart Research Center Systems Biology, University of Stuttgart, Stuttgart, Germany; 8 Experimental Dermatology, Department of Dermatology, Technical University Dresden, Dresden, Germany; 9 Stuttgart Centre for Simulation Science (SC SimTech), University of Stuttgart, Stuttgart, Germany; 10 Centre for Systems Medicine, Royal College of Surgeons in Ireland, Dublin, Ireland

**Keywords:** Tumour biomarkers, Cell biology

## Abstract

Second generation TRAIL-based therapeutics, combined with sensitising co-treatments, have recently entered clinical trials. However, reliable response predictors for optimal patient selection are not yet available. Here, we demonstrate that a novel and translationally relevant hexavalent TRAIL receptor agonist, IZI1551, in combination with Birinapant, a clinically tested IAP antagonist, efficiently induces cell death in various melanoma models, and that responsiveness can be predicted by combining pathway analysis, data-driven modelling and pattern recognition. Across a panel of 16 melanoma cell lines, responsiveness to IZI1551/Birinapant was heterogeneous, with complete resistance and pronounced synergies observed. Expression patterns of TRAIL pathway regulators allowed us to develop a combinatorial marker that predicts potent cell killing with high accuracy. IZI1551/Birinapant responsiveness could be predicted not only for cell lines, but also for 3D tumour cell spheroids and for cells directly isolated from patient melanoma metastases (80–100% prediction accuracies). Mathematical parameter reduction identified 11 proteins crucial to ensure prediction accuracy, with x-linked inhibitor of apoptosis protein (XIAP) and procaspase-3 scoring highest, and Bcl-2 family members strongly represented. Applied to expression data of a cohort of *n* = 365 metastatic melanoma patients in a proof of concept in silico trial, the predictor suggested that IZI1551/Birinapant responsiveness could be expected for up to 30% of patient tumours. Overall, response frequencies in melanoma models were very encouraging, and the capability to predict melanoma sensitivity to combinations of latest generation TRAIL-based therapeutics and IAP antagonists can address the need for patient selection strategies in clinical trials based on these novel drugs.

## Introduction

The immune system can eliminate cancer cells by activating cell surface apoptosis-inducing death receptors, such as tumour necrosis factor-related apoptosis-inducing ligand receptors 1 and 2 (also known as death receptors 4 and 5 (DR4/5)). Many cancer cells, including melanoma, over-express these TRAIL-Rs, possibly due to an additional role these receptors can play in supporting cellular proliferation and invasion by autonomous TRAIL/TRAIL-R signalling [1]. Developing TRAIL-based therapeutics has been a highly active but only moderately successful translational research field for many years, but recent progress in designing superior TRAIL-based biologics and an improved mechanistic understanding of drug-induced TRAIL-sensitisation now provide novel avenues for new anti-cancer therapies [2]. Latest generation TRAIL-derived therapeutics overcome limitations of previous formulations by significantly improving TRAIL receptor oligomerisation and activation by higher valency, and by exerting significantly prolonged serum half-lives. Highly promising variants are hexavalent fusion proteins that couple two single-chain TRAIL trimers and that outperform soluble human TRAIL and TRAIL-R-targeting antibodies [3–5]. Cellular inhibitor of apoptosis proteins (cIAPs) 1 and 2 can prevent TRAIL-induced cell death by recruiting components of the linear ubiquitin chain assembly complex (LUBAC) to aggregated TRAIL-Rs. The activitiy of LUBAC promotes pro-survival signalling and suppresses both apoptosis and necroptosis signalling cascades [6]. Synthetic IAP antagonists, such as Birinapant (TL32711), BV6 or LCL-161, therefore potently sensitise cells to TRAIL-induced caspase-8 activation and apoptosis [7, 8]. IAP antagonists bind to cIAPs and cause conformational changes that allow dimerisation of cIAP RING domains, auto-ubiquitylation and subsequent proteasomal degradation [9]. In cells capable of activating caspase-8, the cleavage of the Bcl-2 family protein Bid initiates the formation of Bax/Bak pores in the outer mitochondrial membrane, followed by activation of downstream caspases-9, -3, -7 and subsequent cell death [10]. Birinapant also binds to and inhibits x-linked inhibitor of apoptosis protein (XIAP), a major antagonist of caspases-9, -3, -7 that is also involved in upstream regulation of cell death signalling, with nM affinity [11–13]. Inducing apoptosis through the TRAIL pathway can proceed without the need for transcriptional responses or protein neo-synthesis, processes required for cell death induction by the majority of cytotoxic therapeutics. This suggests that pre-treatment amounts of proteins regulating apoptotic TRAIL signalling might suffice to derive predictors for treatment responsiveness.

Especially in highly heterogeneous cancers, such malignant melanoma, predictive markers and validated companion diagnostic tests developed from such markers will be necessary to identify those patients likely to respond to treatment [14, 15]. The incidence of cutaneous melanoma continues to rise rapidly [16]. While chemotherapy-based treatments provide little benefit for patients with metastatic melanoma, more recent treatment options such as targeted immuno-therapeutics, BRAFV600 and MEK inhibitors, and combinations thereof in many cases can prolong survival or, less frequently, induce lasting disease remission [17, 18]. However, substantial numbers of patients do not qualify for these treatments or experience disease relapse, so that additional treatment options, for example those building on TRAIL-based therapeutics and IAP antagonists, can be attractive alternatives should it become possible to reliably predict treatment responsiveness.

Here we can report that expression profiles of TRAIL pathway regulators can serve to predict responsiveness to the combination of IZI1551, a prototypical example of a translationally relevant latest generation TRAIL-based biologic [3], and Birinapant (TL32711), a well-characterised example for a translationally relevant IAP antagonist [8]. Across a diverse and heterogeneous melanoma cell line panel, 3D multi-cellular tumour spheroids (MCTS) and melanoma cells isolated from patient metastases, we achieved >80% prediction accuracy. A proof of concept in silico trial based on a cohort of 365 metastatic melanoma patients indicates that IZI1551/Birinapant responsiveness could be expected for up to 30% of tumours.

## Materials and methods

### Materials

TL32711 (Birinapant) was obtained from Active Biochem, Germany. IZI1551 was produced and purified as described before (Hutt et al. 2017). Q-VD-OPh was bought from Selleckchem, Germany. cIAP1 and cIAP2 recombinant proteins, required to determine absolute expression amounts in melanoma cells, were bought from R&D, Germany.

### Melanoma cell lines and freshly isolated melanoma cells

Melanoma cell lines SkMel5 (ATCC; HTB-70), Malme 3M (ATCC; HTB-64), SkMel2 (ATCC; HTB-68), SkMel147 (Memorial Sloan Kettering Cancer Center; NY), WM3060 (Wistar; WC00126), WM1791c (Wistar; WC00086), MeWo (ATCC; HTB-65), Mel Juso (DSMZ; ACC74), WM1366 (Wistar; WC00078), WM115 (ATCC; CRL-1675), WM35 (Wistar; WC00060), WM3211 (Wistar, WC00045), WM793 (Wistar, WC00062), WM852 (Wistar, WC00065), WM1346 (Wistar, WC00121) and WM3248 (Wistar, WC00081) were purchased from ATCC (Mannasas, VA, USA), DSMZ (Braunschweig, Germany) or the Wistar Institute (Philadelphia, PA, USA). Six cell lines carried activating BRAF mutations (WM35, WM793, WM3248, WM115, SkMel5 and Malme 3M), six cell lines NRAS mutations (WM1366, WM1346, SkMel147, SkMel2, Mel Juso, WM3060), one cell line a CDK4 mutation (WM1791c), one cell line carried a c-KIT mutation (WM3211), one cell line carried both NRAS and BRAF mutations (WM852) and one cell line was BRAF/NRAS/c-KIT/CDK4 wildtype (MeWo). All cell lines were purchased as authenticated STR-profiled stocks directly from the vendors. Freshly isolated melanoma cells (M10, M20, M32, M34, M45) were obtained from metastases and prepared for experiments by the Department of Dermatology, University of Dresden, Germany. Two metastases carried BRAF activating mutations (M10 and M45), while three carried activating NRAS mutations (M20, M32 and M34). Further materials (M51_1, M52_2 and M54) were obtained for extended validation (M54, BRAF/NRAS wildtype; M51_1, M51_2 carried BRAF activating mutations). Cell isolates were obtained as part of routine resections at University Hospital Dresden, under the auspices of the local Ethics Committee (ethical approval number EK335082018). Informed consent was obtained from all subjects. Cells were maintained in RPMI-1640 (Thermo Fisher Scientific, Germany) supplemented with 10% (v/v) FBS Brazil One (PAN Biotech, Germany) at 37 °C and 5% CO_2_. Mycoplasma testing was regularly conducted.

### Culturing of 3D spheroids

Cells were harvested and diluted to the concentration of 10^4^ cells/mL in RPMI-1640/10% FBS with the addition of 0.24% Methyl Cellulose (Sigma Aldrich, Germany). 250 cells per drop were placed into the lid of a Petri dish filled with PBS. Spheroids were incubated for 10 days at 37 °C and 5% CO_2_. The medium was exchanged every other day. Slower growing Malme 3M cells and freshly obtained metastatic melanoma cells (M34) were seeded at 500 cells per drop and incubated for 2 weeks.

### Flow cytometry

#### Semi high-throughput cell death measurements

Cells were washed, trypsinised and stained with propidium iodide (PI, Sigma Aldrich, Germany) at 1.33 µg/mL for 10 min. The measurements were performed on a high throughput flow cytometer (BD LSRII SORP) using the 488 nm laser for excitation, while emission was recorded at 617 nm. Flow cytometry data were analysed using Cyflogic v. 1.2.1 (CyFlo Ltd, Finland). All experiments were performed in triplicates and in *n* = 3 independent repeats.

#### Annexin V-GFP or APC/PI staining

Cells were harvested and washed in PBS and Annexin V Binding buffer (Biolegend, Germany). Cells were stained with Annexin V-APC (Biolegend, Germany) (0.1%) or Annexin V-GFP (made in-house, 0.1%) and PI (Biolegend, Germany) (1 µg/mL). Measurements were conducted on a BD FACS Canto II flow cytometer using 561 nm excitation (emission from 600 to 620 nm) (PI) or 640 nm excitation (emission from 655 to 685 nm) (APC). Alternatively, measurements were conducted with a MacsQuant flow cytometer using 488 nm excitation (emission from 655 to 730 nm (PI), and emission from 500 to 550 nm (GFP)). Flow cytometry data were analysed either with the BD FACS Diva software (BD Biosciences, USA) or with Flowing software (Turku Centre for Biotechnology, Finland).

#### TRAIL receptor measurements

Cells were harvested and blocked in ice-cold PBA buffer (1 × PBS, 0.25% BSA and 0.02% Sodium Azide). Surface death receptors were probed with the following antibodies for 1 h at 4 °C: mouse anti-TRAIL R1/TNFRSF 10A (1:100, R&D Systems), mouse anti-TRAIL R2/TNFRSF 10B (1:100, R&D Systems), mouse anti-TRAIL R3/TNFRSF 10C (1:100, R&D Systems) mouse anti-TRAIL R4/TNFRSF 10D (1:100, R&D Systems), purified mouse IgG1 (1:100, R&D Systems) and purified mouse IgG2b (1:100, R&D Systems). Secondary goat anti-mouse FITC conjugated antibody (1:50, Dako, Biozol, Germany) was added for 45 min at 4 °C. Cells were analysed in a MacsQuant flow cytometer using 488 nm excitation (emission was recorded at 500-550 nm). The surface expression of death receptors was calculated by calibration against quantification beads (QIFIKIT, Biozol, Germany), comparing the mean FITC signal of cells to calibration signals. Data were analysed with Flowing Software.

### Western blot analysis

#### Protein quantification

Cells were trypsinised, washed in PBS, centrifuged and lysed in lysis buffer (150 mM NaCl, 1 mM EDTA, 20 mM TRIS, 1% Triton x-100, pH = 7.6) with addition of phosphatase inhibitor (PhosSTOP, 20×, Roche, Germany) and protease inhibitor cocktails (cOmplete, 20×, Roche, Germany). Spheroids were additionally sonicated. The total protein concentration was determined with Bradford assay. 20 µg of protein were resolved on Nu-PAGE^TM^ 4–12% Bis–Tris Midi gels (Invitrogen, Thermo Fisher Scientific, Germany) at 200 V, 400 mA for 40 min, followed by transfer to nitrocellulose membranes using an iBlot device (Invitrogen, Thermo Fisher Scientific, Germany). The membranes were blocked in 0.5× Blocking Solution (Roche, Germany) for 1 h at room temperature. The following primary antibodies were used for overnight incubations at 4 °C: mouse anti-Apaf-1 (1:1000; BD Transduction Laboratories), rabbit anti-Bak (1:1000; CST), rabbit anti-Bax (1:1000, CST), mouse anti-Bcl2 (1:1000; BD Transduction Laboratories) rabbit anti-Bcl-xL (1:1000, CST), mouse anti-Bid (1:1000, BD Transduction Laboratories), rabbit anti-Caspase 3 (1:1000; CST), mouse anti-Caspase 8 (1:1000; CST), rabbit anti-Caspase 9 (1:1000, CST), rabbit anti-cIAP1 (1:1000, Abcam), rabbit anti-cIAP2 (1:1000, Abcam), mouse anti-cFLIP (1:500, Abcam), mouse anti-cFLIP (1:500, Enzo), mouse anti-Cytochrome C (1:1000, BD Transduction Laboratories), rabbit anti-FADD (1:1000, Santa Cruz), rabbit anti-Mcl1 (1:1000, CST), mouse anti-PARP (1:1000, BD Transduction Laboratories), mouse anti-SMAC/DIABLO (1:1000, BD Transduction Laboratories), mouse anti-XIAP (1:1000, BD Transduction Laboratories), mouse anti-XIAP (1:1000, CST), mouse anti-actin (1:10000, CST). Subsequently, membranes were washed 3 × 10 min in TBST and incubated with secondary antibody (goat anti-rabbit IRDye 680 (1:10000) or goat anti-mouse IRDye 800 (1:5000) (LI-COR Biosciences) for 15 min at room temperature, followed by 10 min washing with TBST. Signals were captured on an Odyssey LiCor Imaging System. The quantification of proteins was performed on raw 16 bit images using Odyssey V3.0 software (LI-COR Biosciences). The intensities of the fluorescent signals were corrected for loading.

### Data processing and analysis for predictor identification

All data processing and analysis were performed using a customised version of a previously developed pipeline [19]. The script was developed for MATLAB 2017b (The Mathworks, UK), equipped with the statistical toolbox. Prior to statistical analysis, protein data were mean-centred and scaled, dividing by the respective standard deviation. A principal component analysis (PCA) was performed on the standardised dataset and the PCs with an eigenvalue >1 were used for subsequent analyses. Linear discriminant analysis (LDA) was applied to objectively assess the accuracy of response class separation in the space defined by the first six PCs. Then, leave-one-out cross-validation (LOOCV) was applied iteratively to the 16-cell line panel to assess predictive capacity. For each iteration, data from 15 cell lines were used as a training set to define the PC space, and one test cell line was subsequently positioned according to its protein expression profile. LDA was then applied to determine if the test cell line was placed in the correct responsiveness sub-space. The response of 3D grown and patients-derived primary cell lines was predicted with the same workflow, using the predictor obtained from the data set of the 16-cell lines panel. The optimal predictive protein subset (reduced predictor) was determined using the *Select attributes* panel of the WEKA workbench (Version 3.8.2 [20]). A ranking of the proteins was obtained using the *CorrelationAttributeEval* attribute evaluator with *Ranker* search method and 10-fold cross-validation mode. This attribute selection method evaluates the *merit* of each protein individually by calculating the Pearson’s correlation between the individual protein and the responsiveness class. The attribute selection step was performed using the proteins quantified in the 2D cell lines panel. The complete prediction pipeline was iteratively applied taking into account the first six PCs, and removing the protein with the lowest rank at each iteration. Statistical analyses not described above were performed with GraphPad Prism 7 (GraphPad Software).

### In silico trial

The protein expression patterns of the melanoma cell line panel were used to estimate the protein expression profiles in melanoma tumours of 472 patients for which transcriptome data are deposited in the cancer genome atlas melanoma cohort (TCGA-SKCM). Normalised mRNA expression data (Upper Quartile normalised Fragments per Kilobase of transcript per Million mapped reads, log2(FPKM-UQ+1)) generated by the Genomic Data Commons (GDC-NIH) were downloaded from the UCSC-XENA browser (Available at: https://xena.ucsc.edu/. Accessed: 4 February 2019). Data interpolation was performed using *Point-to-point* curve creation in GraphPad Prism 7 (GraphPad Software). Standard curves were generated using minimum and maximum values of protein expression range (cell line panel) and TCGA-SKCM back transformed mRNA expression data. For response predictions, PCA was applied to the data for the *n* = 11 predictor proteins in the cell lines dataset, followed by LDA-based definition of responsiveness and resistant subspaces, and subsequent positioning of *n* = 365 TCGA derived melanoma metastases in the PC space according to their estimated protein values.

## Results

### IAP antagonist Birinapant sensitises a subset of melanoma cell lines to apoptosis induced by the 2nd generation TRAIL-based biologic IZI1551

**Fig. 1:**
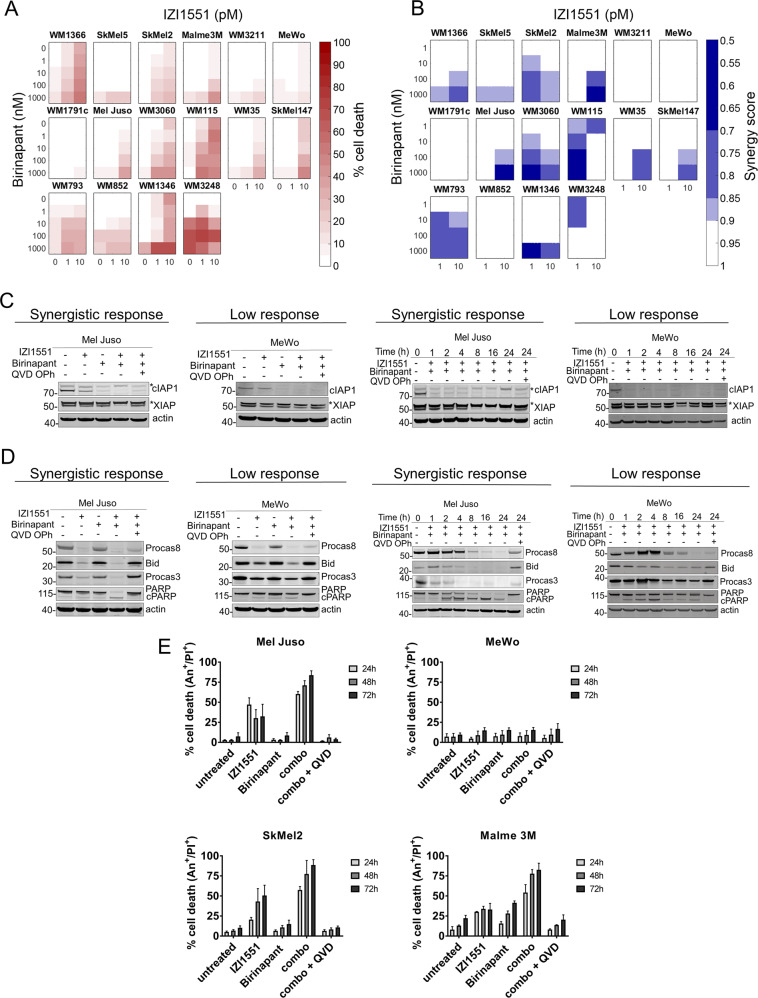
IAP antagonist Birinapant sensitises a subset of melanoma cell lines to IZI1551-induced apoptosis. **a** Melanoma cell lines respond heterogeneously to single and combination treatment of IZI1551 and Birinapant. Cells were treated for 72 h followed by flow cytometric determination of cell death (propidium iodide positivity). Data shown are means from *n* = 3 independent experiments. **b** Synergy scores for treatment combinations, as calculated by Webb’s fractional product method. **c** Treatment-induced changes in IAP amounts, analysed by Western blotting. Actin served as loading control. Asterisks indicate unspecific bands. Representative results from *n* = 3 independent experiments are shown. **d** Apoptotic signalling was studied 24 h after single and combination treatment with IZI1551, Birinapant and Q-VD-OPh (30 µM). Actin served as a loading control. Representative results from *n* = 3 independent experiments are shown. **e** Melanoma cell lines die by apoptosis upon combination treatment. Cell lines were treated with 1 nM IZI1551, 1 µM Birinapant, with or without 30 µM Q-VD-OPh. Cells were stained with PI and Annexin V-APC and analysed by flow cytometry. Shown are mean values + SD of three independent experiments.

To study the responsiveness and the response heterogeneities of melanoma cells to IZI1551, a novel and translationally relevant hexavalent TRAIL receptor agonist [3], to the IAP antagonist TL32711/Birinapant, a compound currently evaluated in clinical trials [21], or combinations thereof, we employed a diverse set of sixteen cell lines (see materials and methods). For each cell line, cell death was determined at 15 treatment conditions, using semi-high throughput flow cytometry. Cell lines varied in their response to the treatments, ranging from high resistance to high sensitivity (Fig. 1a). Many cell lines responded synergistically to the combination treatment (synergistic responders; WM1366, SkMel5, SkMel2, Malme3M, Mel Juso, WM3060, WM115, WM35, SkMel147, WM793, WM1346, WM3248), as determined using Webb’s fractional product method, whereas others (WM3211, MeWo, WM1791c, WM852 cells) failed to do so (low responders) (Fig. 1b).

Birinapant had on-target activity in both synergistic responders and low responders, since cIAP1 protein amounts were efficiently and rapidly lost upon single agent and combination treatments (Fig. 1c). Neither single nor combination treatment induced detectable amounts of TNFα secretion (not shown), a response to IAP antagonists that in rare cases can contribute to autocrine cell death induction [22]. The amounts of XIAP remained largely unchanged, except for the combination treatment in synergistically responding Mel Juso cells (Fig. 1c). XIAP is a known caspase-3 substrate [23], and correspondingly caspase inhibitor Q-VD-OPh restored XIAP amounts, indicating that IZI1551/Birinapant induces apoptosis in responder cell lines such as Mel Juso (Fig. 1c). This was further supported by the processing of procaspases 8 and 3, and by the caspase-dependent cleavage of Bid and PARP in Mel Juso cells (Fig. 1d). In poorly responding MeWo cells, instead, PARP cleavage was modest and detectable only as a transient pulse (Fig. 1d, e). In line with these observations, caspase inhibitor Q-VD-OPh prevented IZI1551 and IZI1551/Birinapant induced cell death in Mel Juso cells and other synergistic responders, such as SkMel2 and Malme 3M (Fig. 1e).

Taken together, these results show that Birinapant sensitises a subset of human melanoma cell lines to cell death induced by IZI1551, a 2nd generation TRAIL-based therapeutic, and that apoptosis appears to be the primary cell death modality in synergistic responders.

### Expression patterns of apoptosis proteins allow predicting IZI1551/Birinapant responsiveness

**Fig. 2:**
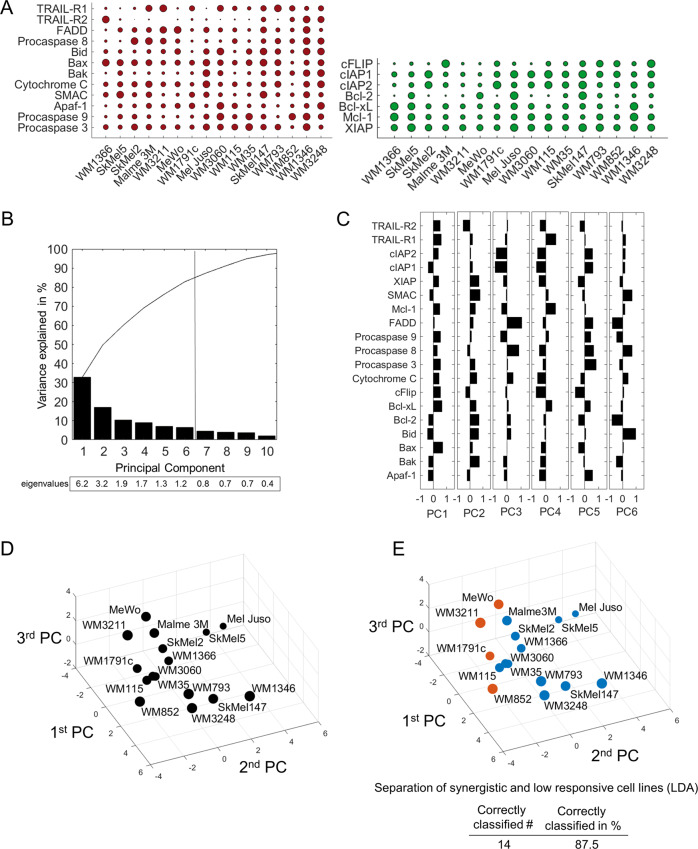
Expression patterns of apoptosis proteins separate resistant from synergistically responding cell lines. **a** Baseline expression of pro- and anti-apoptotic proteins of the TRAIL pathway. Circles summarise 684 quantifications, and circle sizes represent relative expression amounts of the proteins between cell lines. Protein amounts are provided in Supplemental Table 1. **b** Percentage of the variance of the original dataset explained by PCs. PCs with an eigenvalue >1 were retained for further analysis. Accumulated “variance explained” is plotted in black. **c** Weight coefficient table. Bars represent the contributions of the respective proteins to the different PCs. **d** Cell lines positioned in a multidimensional space according to their individual protein expression profiles. The PC space shown was defined by the first three PCs. Circle sizes decrease with distance from the observer to aid 3D visualisation. **e** Colour coding indicates responsiveness of cell lines to IZI1551/Birinapant (orange = low response; blue = synergistic response). Table insert indicates accuracy of spatial segmentation between low and synergistic responders.

The combination of IZI1551/Birinapant can induce apoptotic cell death without the need for protein neo-synthesis. We therefore next explored if baseline expression amounts of apoptosis proteins carry information on the responsiveness of melanoma cell lines to the combination of IZI1551/Birinapant. Pre-treatment amounts of 19 key pro- and anti-apoptotic players that regulate the apoptotic TRAIL signalling pathway was determined by quantitative immunoblotting at high dynamic range or, for death receptors, by cell surface staining (Fig. 2a; Supplemental Fig. 2). Expression patterns varied considerably between the proteins and across the cell lines. To explore possible correlations between protein expression patterns, we conducted a PCA. A total of six principle components (PCs), all with an eigenvalue >1 and thus fulfilling the Kaiser criterion [24], were required to capture approximately 80% of the data variance (Fig. 2b), highlighting that pre-treatment expression patterns were highly heterogeneous. Similarly, the associated weight coefficients indicated that individual proteins contributed heterogeneously to the first six PCs, without obvious positive or negative correlations between pro- and anti-apoptotic proteins (Fig. 2c). A visualisation of the cell line positions within the space defined by the first three PCs correspondingly failed to identify visually distinct clusters of cell lines (Fig. 2d). In conclusion, these data demonstrate high expression heterogeneity between proteins and between the cell lines.

Interestingly, colour coding the cell lines according to synergistic or low responsiveness indicated that synergistically responding and poorly responding cell lines occupy distinct regions within the plotted space (Fig. 2e). LDA confirmed this visual impression, with 14/16 cell lines (88%) correctly separated into their respective response categories. These results, therefore, indicate that even though apoptosis protein expression is highly heterogeneous across the cell lines, the expression patterns nevertheless carry information on the capability to respond synergistically to the combination of IZI1551/Birinapant.

**Fig. 3:**
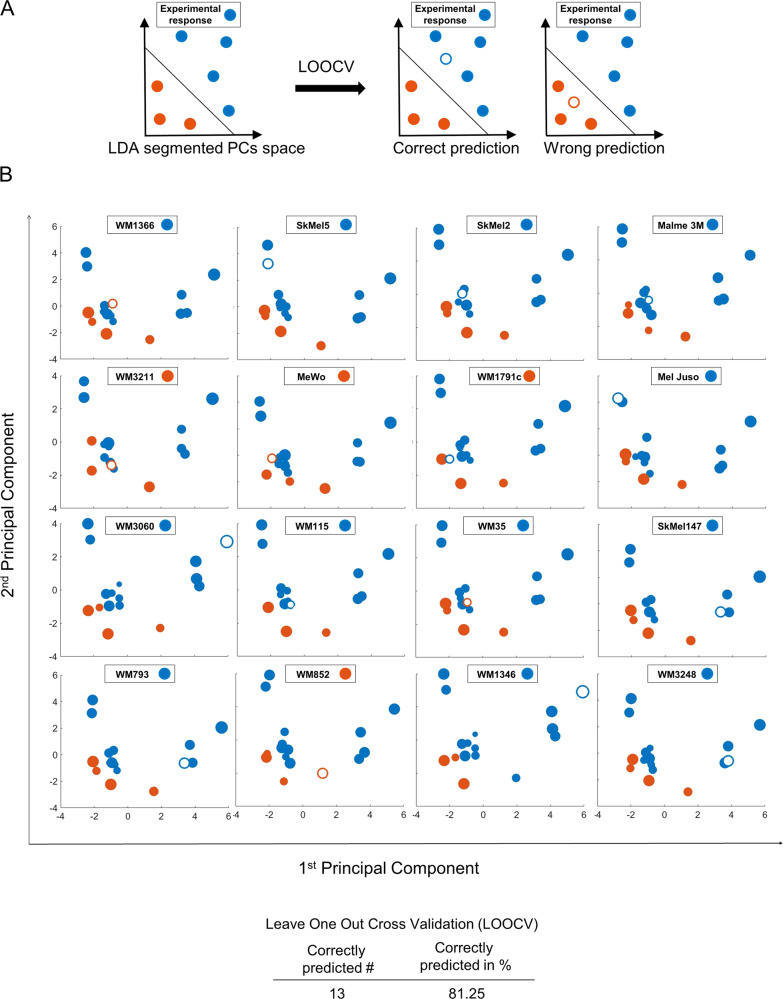
Expression patterns of apoptosis proteins allow predicting IZI1551/Birinapant responsiveness. **a** Simplified 2D schematic showing the workflow for determining prediction accuracy by combined PCA/LDA/LOOCV. Following PCA, an LDA separates the PC space into areas for synergistic responsiveness and low responsiveness. A cell line of unknown responsiveness (empty circle) is then placed into the segmented PC space according to its protein expression profile, with the positioning serving as the response prediction. Experimental responsiveness data served to validate predictions. **b** 2D projection of LOOCV results for the 16 cell lines. The responsiveness of the test cell line was predicted (blue for synergistic, orange for low responsive). The empty circle represents the test cell line being placed into the PC space. Circle sizes decrease with distance from the observer to aid 3D visualisation. Table insert summarises prediction accuracy.

We next tested if the protein expression patterns would be sufficient to predict responsiveness or resistance to IZI1551/Birinapant in melanoma cell lines. To this end, we performed LOOCV based on the approach described above. PCAs were conducted for sets of 15 cell lines, followed by LDAs to define the hyperspace regions of responsiveness and resistance. Missing cell lines were subsequently positioned into the LDA-segmented PC spaces according to their individual expression patterns of apoptosis regulators. If the tested cell line positioned into the correct response region, the prediction was considered successful (Fig. 3a). Overall, LOOCV was sufficient to correctly predict the responsiveness of 13 out of 16 cell lines (81%) (Fig. 3b), indicating that the measured protein panel allows predicting responsiveness to IZI1551/Birinapant on a case-by-case basis with high accuracy.

### Responses to IZI1551/Birinapant can be predicted for 3D growth conditions

**Fig. 4:**
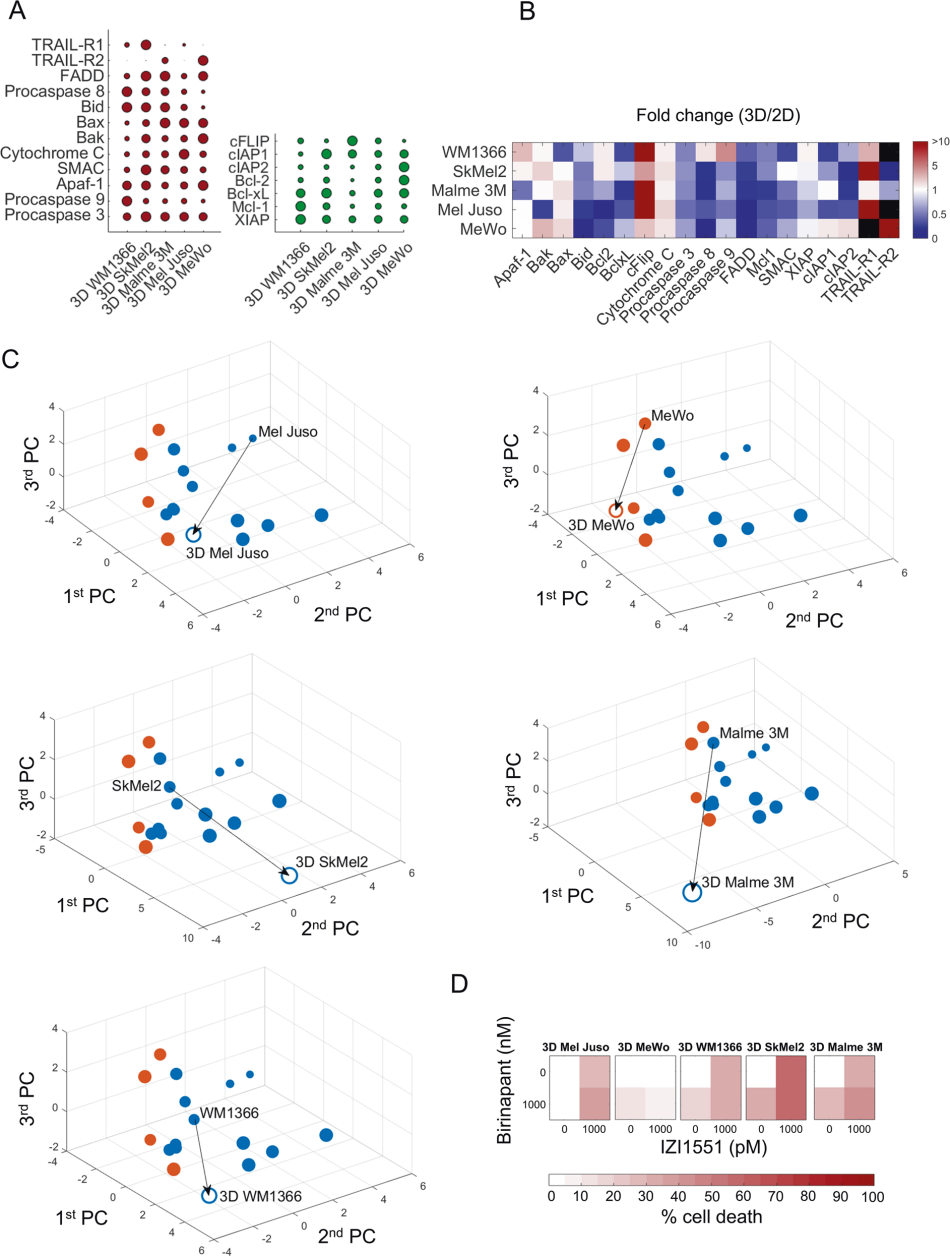
Responses to IZI1551/Birinapant can be predicted for 3D growth conditions. **a** Quantification of pro- and anti-apoptotic proteins in cell lines grown as MCTS (red and green, respectively). Circles summarise 285 quantifications and circle sizes represent mean protein quantities determined from at least *n* = 3 independent experiments. Protein amounts are provided in Supplemental table 1. **b** Heatmap showing the fold change in protein expression between 3D and 2D culture. Black colour indicates absence in either 2D or 3D conditions. **c** Positioning of cell lines grown in 3D in the PC space defined by 2D cultured cell lines. Empty circles indicate positions of cell lines grown in 3D. Arrows indicate the change of position in the PC space caused by altered protein expression between 2D to 3D growth conditions. Circle colours reflect expected responsiveness (blue) or resistance (orange), based on the LDA segmented PC space. The circle size decreases with distance from the observer to aid 3D visualisation. **d** Experimental validation of MCTS responsiveness to IZI1551/Birinapant treatment. MCTS of cell lines were treated with IZI1551 (1 nM) and Birinapant (1 µM) or their combination for 24 h. Cell death was measured by flow cytometry (PI uptake). Data show means of *n* = 3 measurements.

We next studied if responsiveness to IZI1551/Birinapant can be predicted for cells grown as MCTS. While more demanding as a cell culturing method, spheroids provide the advantage of higher microenvironmental complexity at nevertheless well-controlled experimental conditions [25]. Protein quantification from spheroids of five cell lines able to form MCTS demonstrated that the transition from 2D cell culture to 3D spheroid culture substantially affected protein expression patterns (Fig. 4a, b, Supplemental Fig. 3). A number of pro- as well as anti-apoptotic proteins were considerably downregulated, such as Bid, Bcl-2, Procaspase 3, FADD and Mcl-1. cFLIP and TRAIL-R1, instead, appeared to accumulate, and a number of other proteins changed heterogeneously in their expression amounts across spheroids of different cell lines (Fig. 4b). While a reductionist reasoning based on individual protein changes would intuitively suggest that IZI1551/Birinapant responsiveness of 3D MCTS should differ from 2D cultures, the combined complexity of altered protein expression prevents drawing conclusions prior to experimental validation. We therefore used the PCA/LDA-based approach to generate testable predictions on MCTS responsiveness. Positioning the MCTS forming cell lines into the PC space according to their respective pathway proteome revealed that their coordinates differed substantially from their 2D cultivated counterparts (Fig. 4c). Interestingly, despite the substantial changes in relative protein amounts, all cell lines were predicted to remain within their respective response class (Fig. 4c, colour-coded open circles). To test these in silico predictions, we measured cell death in spheroids treated with IZI1551, Birinapant or the combination thereof. Indeed, the predictions could be confirmed for all five cell lines, with SkMel2, WM1366, Mel Juso and Malme 3M responding to the combination treatment of IZI1551/Birinapant, and MeWo cells remaining resistant in the 3D growth scenario (Fig. 4d). TNFα was not secreted upon growth in 3D or in response to the treatments, as tested for Mel Juso and MeWo cells (not shown). Overall, we therefore conclude that a PCA/LDA-based prediction framework, parameterised with protein expression and treatment responsiveness data from 2D cell cultures, is sufficient to predict responses to IZI1551/Birinapant for 3D spheroid growth conditions.

### Responses to IZI1551/Birinapant can be predicted for melanoma cells freshly isolated from metastases

**Fig. 5:**
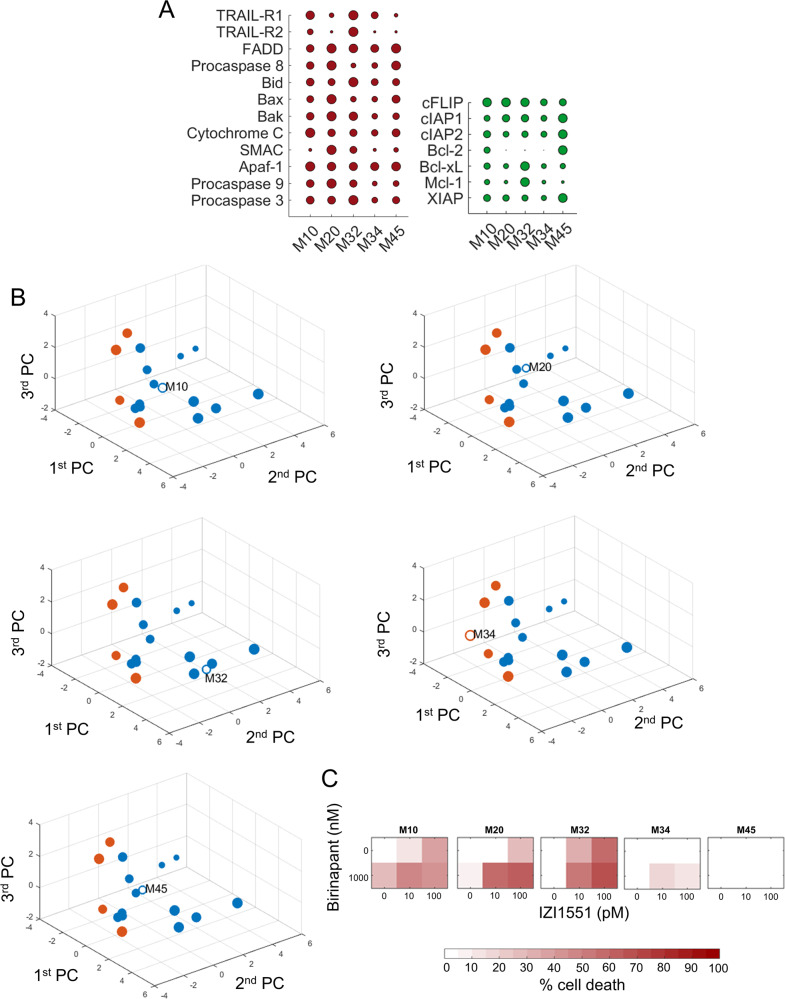
Responses to IZI1551/Birinapant can be predicted for cells isolated from melanoma metastases. **a** Quantification of apoptosis regulatory proteins in cells derived from melanoma metastases. Red coloured circles represent pro-apoptotic and green circles anti-apoptotic proteins. Circles summarise 285 quantifications, and circle sizes represent mean protein quantities determined from at least *n* = 3 independent experiments. Protein amounts are shown in Supplemental table 1. **b** Positioning of melanoma cells from patient metastases in the PC space defined by 2D cultured cell lines. Empty circles indicate positions of patient cells. Circle colours reflect expected responsiveness (blue) or resistance (orange), based on the LDA segmented PC space. The circle size decreases with distance from the observer to aid 3D visualization. **c** Experimental validation of primary melanoma cell responsiveness to IZI1551/Birinapant treatment. Cells were treated as indicated for 24 h. Cell death was measured by flow cytometry (PI uptake). Heat maps show the mean of *n* = 3 independent experiments.

For a translationally more relevant setting, we next tested if IZI1551/Birinapant responses can be predicted for melanoma cells freshly isolated from metastases. Following quantification of apoptosis regulatory proteins (Fig. 5a, Supplemental Fig. 4), cells were positioned into the PC space. Predictions were generated as described above and cells were colour coded according to their expected IZI1551/Birinapant responsiveness. M10, M20, M32 and M45 cells were predicted to respond to IZI1551/Birinapant combination treatment, whereas M34 cells were expected to respond poorly (Fig. 5b). Validation experiments confirmed the predictions on high responsiveness of M10, M32 and M20 cells and poor responsiveness of M34 cells (Fig. 5c). We therefore conclude that high predictions accuracies can also be achieved for cells freshly isolated from clinical materials.

### A reduced predictor maintains performance and estimates response prevalence to IZI1551/Birinapant in metastatic melanoma

**Fig. 6:**
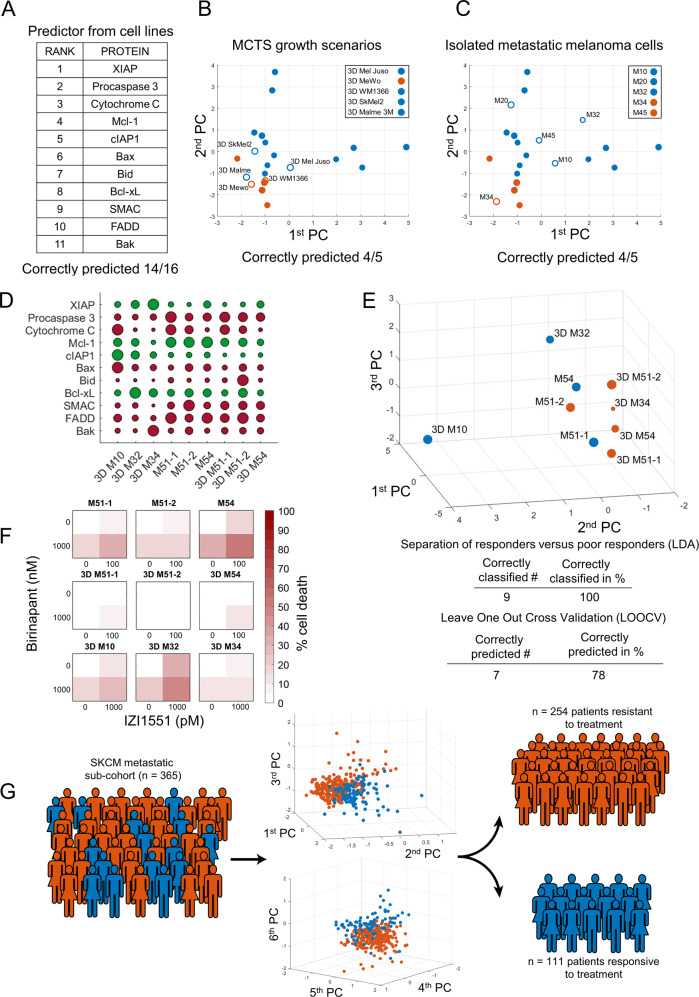
A reduced predictor maintains performance and estimates response prevalence to IZI1551/Birinapant in metastatic melanoma. **a** Ranking of variables in a reduced predictor, as obtained by computed merit. **b**, **c** Responsiveness predictions and prediction accuracies for MCTS growth scenarios and for metastatic melanoma cells isolated from patients. The PC space is shown as a two-dimensional projection. Filled circles represent training data from the melanoma cell line panel. Open circles highlight positions of MCTS (**b**) or cells isolated from melanoma metastases (**c**). **d** Quantities of apoptosis regulators in additional validation samples. Circle sizes represent relative protein amounts. Protein amounts are listed in Supplemental table 1. Western blots are shown in Supplemental Fig. 5. **e** Validation samples positioned in the PC space obtained by the reduced predictor. Colour-coding indicates responsiveness. Table inserts display accuracy of spatial segmentation and prediction accuracy. **f** Experimental responsiveness of validation samples. Cells were treated as indicated for 24 h. Cell death was measured by flow cytometry (PI uptake). Heat maps show the mean of *n* = 3 independent experiments. **g** Estimation of response prevalence in a hypothetical trial. Estimated protein expression profiles of metastatic melanoma patients (*n* = 365) were used to predict responsiveness (blue, *n* = 111) or resistance (orange *n* = 254) to IZI1551/Birinapant combination treatment. 3D graphs show arrangement of predicted responders and non-responders in the predictor space.

The framework to predict responsiveness to IZI1551/Birinapant builds on an otherwise unbiased selection of nineteen regulators known to be involved in canonical apoptosis signal transduction for this treatment combination. We next determined the contribution of the individual protein variables towards accurate predictions. To do so, we used the attribute selection feature of the WEKA workbench [20] to compute the “merit” of each protein, based on the protein expression profiles and the responsiveness data of the melanoma cell line panel. From this, we obtained a ranking of protein variables according to the degree of association with treatment responsiveness (in sequence of decreasing merit: XIAP, Procaspase 3, Cytochrome C, Mcl-1, cIAP1, Bax, Bid, Bcl-xL, Smac, FADD, Bak, cIAP2, TRAIL-R1, Procaspase 9, Apaf-1, TRAIL-R2, Procaspase 8, cFLIP and Bcl-2). We then iteratively performed predictions for the cell line panel, with the protein with the lowest merit removed upon each iteration. Performance was largely maintained (14/16 correct predictions for the cell line panel) when limiting the predictor to the eleven proteins with the highest merit (Fig. 6a). The reduced predictor correctly determined treatment responsiveness in 4/5 MCTS growth scenarios and in 4/5 biopsy-derived fresh melanoma cells (Fig. 6b, c). Further validation of the reduced predictor was conducted using nine additional and independently analysed samples, including three 2D and six 3D growth scenarios. Also in these samples prediction accuracies of approximately 80% were achieved (Fig. 6d–f, Supplemental Fig. 5). Overall, we noted strong influences of XIAP and procaspase-3, direct interactors and regulators of type I signalling competency during extrinsic apoptosis [26, 27], and various members of the Bcl-2 family in the predictor (Fig. 6a). The ability to predict responsiveness to IZI1551/Birinapant in cell lines and ex vivo cultures raises the question if responses can be expected in patients, and if so, how frequent such responses might be. We therefore estimated the clinical response prevalence under the assumption that favourable drug pharmacokinetics and pharmacodynamics allow both drugs to reach their targets. Expression profiles of predictor variables were deduced from transcriptome data of metastatic melanoma patients (*n* = 365, TCGM-SKCM cohort, Supplemental Table 2) by mapping to protein expression ranges measured experimentally. Following positioning into the LDA segmented PC space defined by the predictor, 111 out of 365 patients were expected to respond to treatment (Fig. 6g). The expectation of approximately 30% responders needs to be interpreted in the context of predictor accuracy. The 80% prediction accuracy achieved in the cell line panel is composed of a predictor sensitivity of 92% and a specificity of 75%, so that the predictor strength lies in recalling true positives. Taken together, these results demonstrate that highly accurate predictions can be made for IZI1551/Birinapant responsiveness with a reduced set of input variables, and that in up to 30% of clinical cases an on target responsiveness could be expected, as estimated from a representative cohort of metastatic melanoma patients.

## Discussion

Here, we report that protein expression signatures of TRAIL pathway regulators can serve to predict responsiveness to the combination of IZI1551 and Birinapant, targeted therapeutics with high translational relevance [7, 28]. High accuracies for response predictions were achieved for melanoma cell lines, for 3D multi-cellular melanoma spheroids and for cells newly isolated from melanoma metastases (approximately 80% prediction accuracy). Protein prioritisation resulted in a reduced marker that, when applied in a proof of concept in silico trial, suggests that IZI1551/Birinapant responsiveness could be expected in up to 30% of tumours in patients with metastatic melanoma.

Previous TRAIL-based therapeutics were tested in translational settings and performed unsatisfactorily [28]. Among the reasons for limited efficacy of TRAIL-R agonistic antibodies in the clinic were short serum half-lives and the requirement for immune cell-mediated, Fcγ-dependent clustering of therapeutic antibodies to induce efficient TRAIL-R1/R2 oligomerisation and caspase-8 activation [29]. 2nd generation TRAIL-based therapeutics address these problems, for example by increased valency and by using Fc regions as dimerisation and half-life extension modules [3, 4, 28]. IZI1551, consisting of two tri-valent single-chain TRAIL fragments cross-linked via the Fc part of an IgG antibody, is a prototypical example for this principle and potently induces apoptosis in vivo in cells moderately responsive to traditional TRAIL-based therapeutics [3]. However, in many cases sensitising co-treatments are required to ensure efficient apoptosis induction following TRAIL-R1/R2 activation. IAP antagonists are potent sensitisers to extrinsic apoptosis [21], suppressing the formation of LUBAC and the associated initiation of pro-survival signalling. IAP antagonists also sensitise to apoptosis induced by intrinsic cytotoxic stimuli, such as genotoxic therapeutics in pancreatic, colon and brain cancer [30–32], where cIAPs likely impair caspase-8 binding and activation on cytosolic ripoptosomes [33, 34].

While both 2nd generation TRAIL-R1/R2 agonists as well as IAP antagonists are currently tested in clinical trials (NCT03082209 [5, 21]), currently no studies test their combination. In addition, validated biomarkers predictive of treatment responsiveness do not exist for TRAIL-based therapeutics, IAP antagonists or the combination of both. The lack of reliable molecular markers to predict responses to TRAIL might indeed have contributed to the poor performance of TRAIL-based therapeutics in the clinical setting, since no patient selection could be performed [35]. The absence of response predictors for IAP antagonists likewise affects current clinical trials based on this class of therapeutics [21]. Notably, for both TRAIL-R1/R2 agonists as well as for IAP antagonists, the expression amounts of their direct molecular targets, i.e. TRAIL-R1/R2 amounts and cIAP proteins, appear insufficient to derive response biomarkers [21, 36, 37]. This indicates that treatment efficacy is determined further downstream within the signal transduction network and/or too complex to be captured by traditional or reductionist biomarker discovery approaches.

With IAP antagonists removing the apical suppression of extrinsic apoptosis induction, we hypothesised that the expression amounts of key regulatory proteins of the TRAIL signal transduction network can serve to predict responsiveness. Indeed, predictions on IZI1551/Birinapant responses, based on the expression patterns of key TRAIL pathway regulators, were highly accurate. Being able to predict responsiveness also in a micro-environmentally more complex 3D setting and in cells newly isolated from patients indicates that concerns about using continuously cultured cell lines to develop a predictor for IZI1551/Birinapant responsiveness can be alleviated, possibly because protein expression alone is sufficient to derive treatment responsiveness. Complex genetic characterisations and careful selection of cell line and in vivo models might, however, be warranted for studies on treatment scenarios that are highly dependent on disease-relevant mutations, and accordingly the genetic representation of the disease [38–40].

We initiated our study using 19 proteins considered key regulators of IZI1551/Birinapant induced signal transduction. We could reduce this panel to an 11 protein signature which, compared to traditional biomarkers, still seems rather large. However, this likely reflects the complexity of apoptosis signal transduction and regulation, as well as the disease heterogeneity observed in melanoma. The development of complex protein quantity-based biomarkers for routine clinical application still faces major technological challenges [41, 42]. Traditional immunohistochemical analyses of tumour biopsies typically provides insufficient dynamic range and limited calibration possibilities to derive reliable quantitative data. Alternative approaches, such as reverse phase protein arrays and mass spectrometric analyses of clinical specimen can overcome these hurdles, but are difficult to embed into routine pathology and laboratory workflows in the clinical environment. To take intra-tumour cell-to-cell heterogeneity into account, an aspect likely crucial to refine our predictor in a translational setting, technology such as mass cytometry could provide the possibility to capture multiplexed protein markers at the single cell level [43]. However, this technology is difficult to apply to tissue specimen. Developments in the field of high dynamic range fluorescence-based analysis of FFPE materials, coupled to multiplexing technologies that allow re-staining of tissue slices [44, 45], might more conveniently and routinely allow obtaining quantitative protein expression data, especially where entire cellular proteomes are not required.

It is noteworthy that none of the melanoma models studied lacked TRAIL-R1/R2 or caspase-8 expression, and TRAIL-Rs or caspase-8 amounts did not appear crucial to predict responsiveness. The amounts of these proteins therefore possibly do not limit IZI1551/Birinapant responsiveness in melanoma. A recent study in models of non-small-cell lung cancer and pancreatic ductal adenocarcinoma interestingly indicates that cancer cells might become addicted to TRAIL receptor expression, with autonomous TRAIL-R signalling contributing to disease progression [1]. Additionally, proliferating cells might rely on a cell death-independent role of caspase-8 in contributing to chromosome alignment during mitosis [46]. In the predictor, the expression of XIAP and caspase-3 strongly contributed to accurate response predictions. Both proteins play crucial roles in controlling cellular life/death decisions during apoptosis execution [10, 47]. XIAP additionally holds in check the “type I” link by which caspase-8 can activate caspase-3 [26, 27, 48]. However, kinetically the mitochondrial route still seems preferred in cells capable to die by type I signalling [26], most likely due to the strong amplification of apoptosis signalling by Bcl-2 family dependent mitochondrial outer membrane permeabilisation and apoptosome formation. Indeed, various Bcl-2 family members, such as Mcl-1, Bax, Bid, Bcl-xL and Bak, display prominently in the predictor. Mcl-1 and Bcl-xL negatively regulate Bax/Bak pore formation, while Bid is a primary substrate of both caspase-8 and caspase-3, with truncated Bid inhibiting Mcl-1 and Bcl-xL, and activating Bax and Bak [49]. Taken together, the interplay of caspases-3, XIAP and Bcl-2 family members, initiated by non-limiting amounts of TRAIL receptors and caspase-8, appears to play a central role in melanoma cell death upon exposure to IZI1551/Birinapant.

Taken together, this study represents a successful proof of concept for developing a stratification marker for malignant melanoma in response to a novel, clinically relevant combination treatment based on a 2nd generation hexavalent TRAIL variant (IZI1551) and a representative IAP antagonist, Birinapant. This can form the basis for future translational and clinical studies in which combination treatments of 2nd generation TRAIL-based therapeutics and IAP antagonists will be tested and for which optimal patient selection strategies are required.

## Supplementary information

Supplementary figure 1

Supplementary figure 2

Supplementary figure 3

Supplementary figure 4

Supplementary figure 5

Supplemental table 1

Supplemental table 2

Supplementary Figure and Table Legends
