# Supplementary figures and images for: Convergence of pathway analysis and pattern recognition predicts sensitization to latest generation TRAIL therapeutics by IAP antagonism
Source: Cell Death Differ. 2020 Feb 21;27(8):2417–32. doi: 10.1038/s41418-020-0512-5 (PMC7370234; doi:10.1038/s41418-020-0512-5)

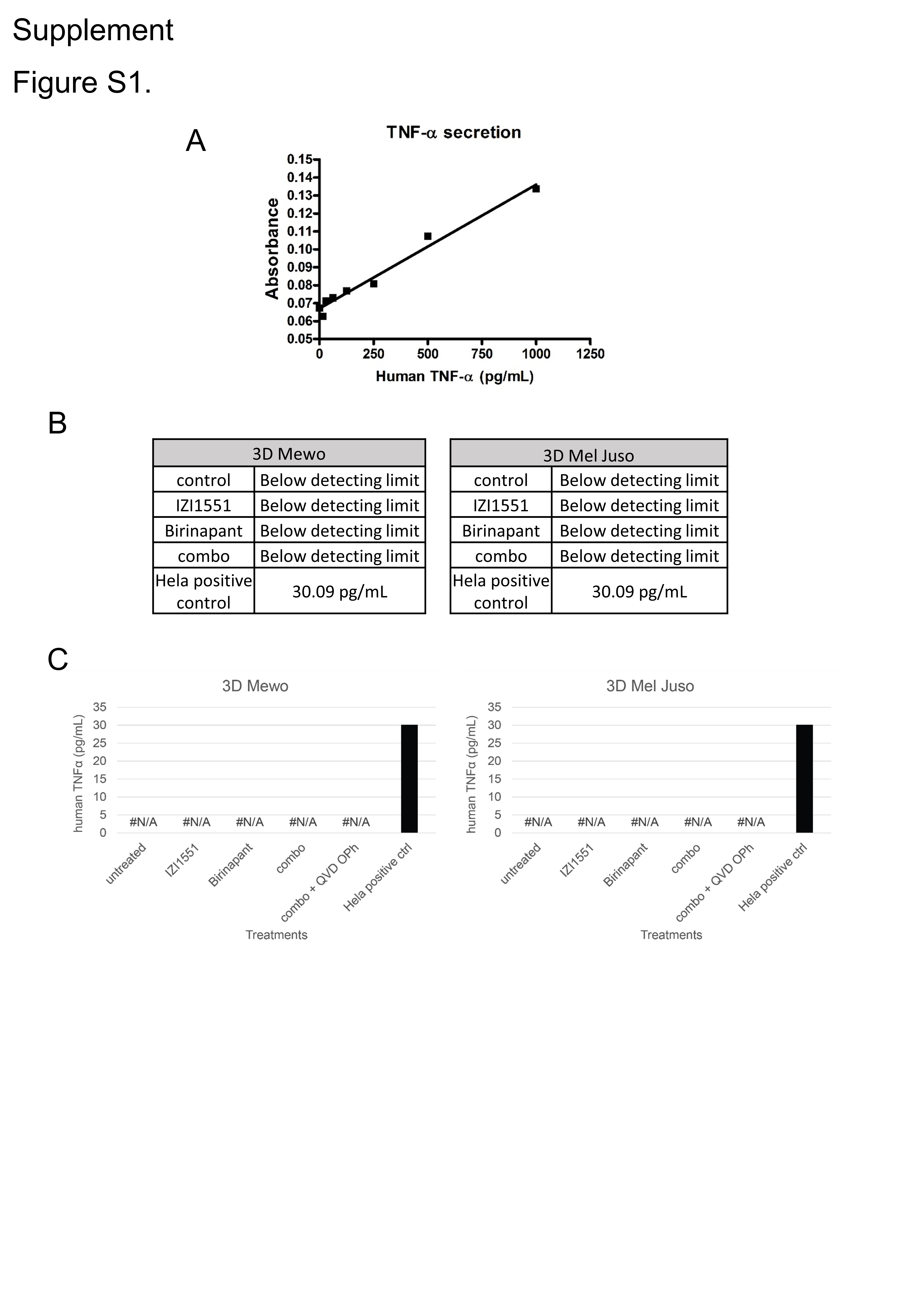

Supplement: Supplementary file 1 — Supplementary figure 1 [file 41418_2020_512_MOESM1_ESM.tif]

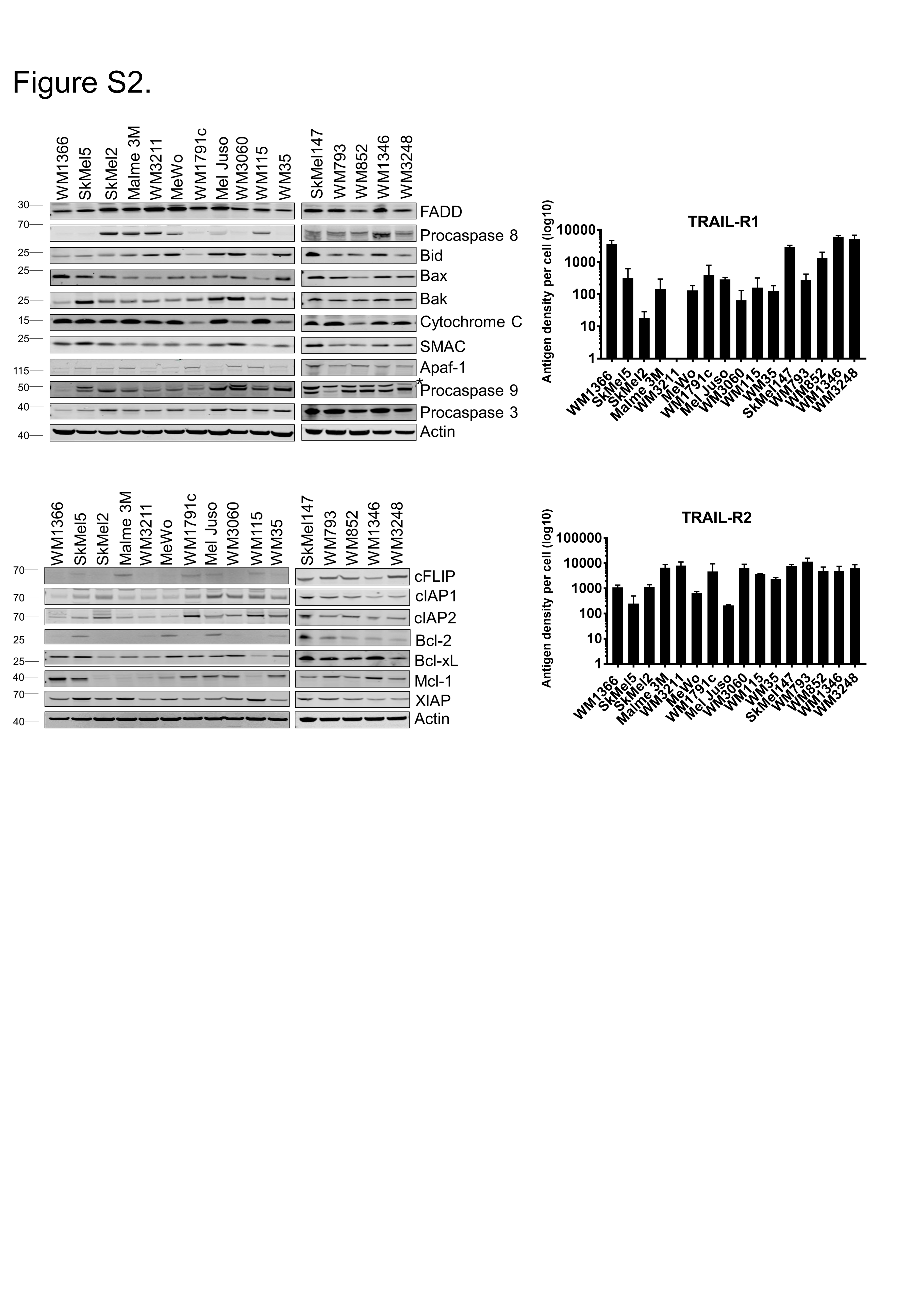

Supplement: Supplementary file 2 — Supplementary figure 2 [file 41418_2020_512_MOESM2_ESM.tif]

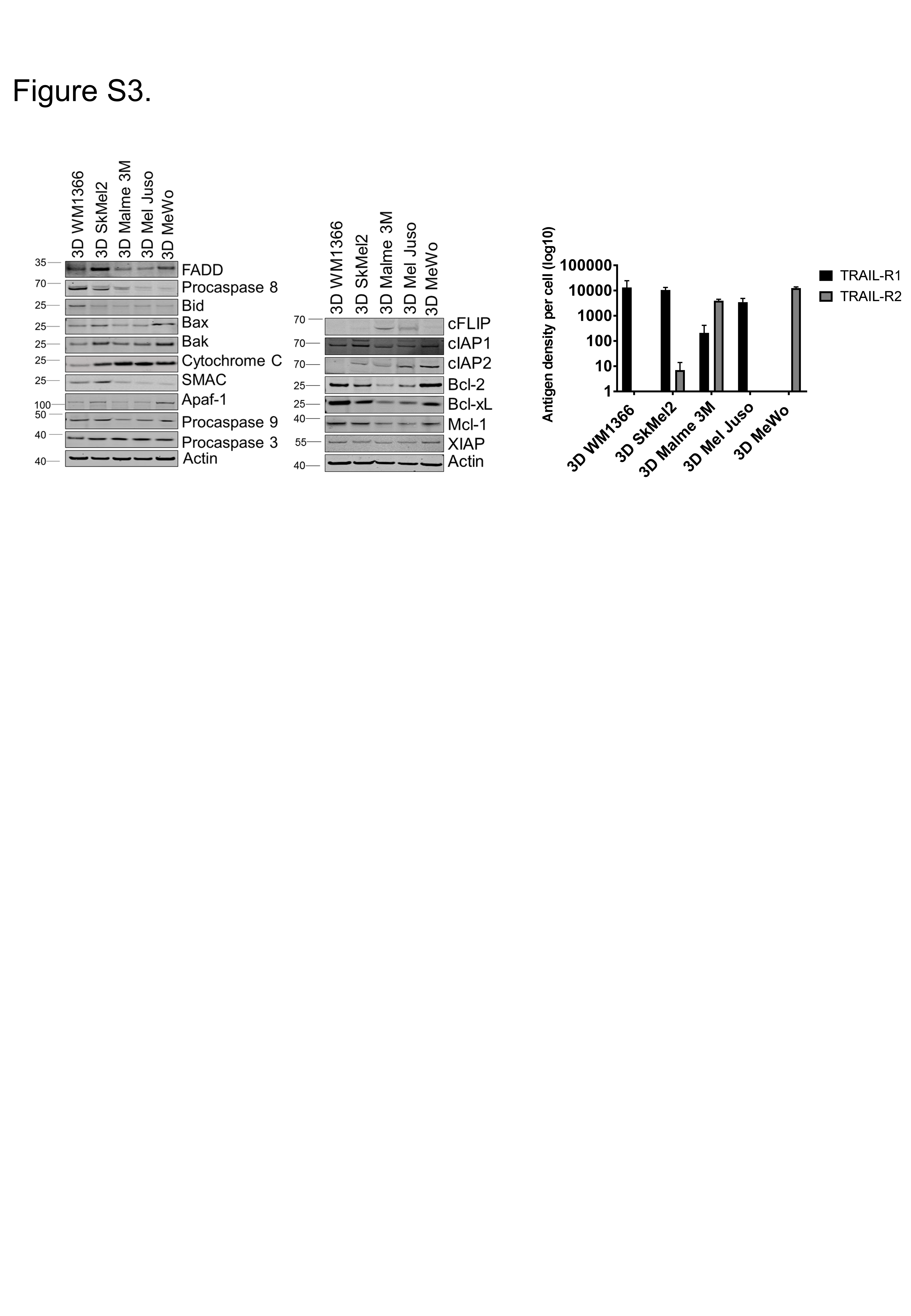

Supplement: Supplementary file 3 — Supplementary figure 3 [file 41418_2020_512_MOESM3_ESM.tif]

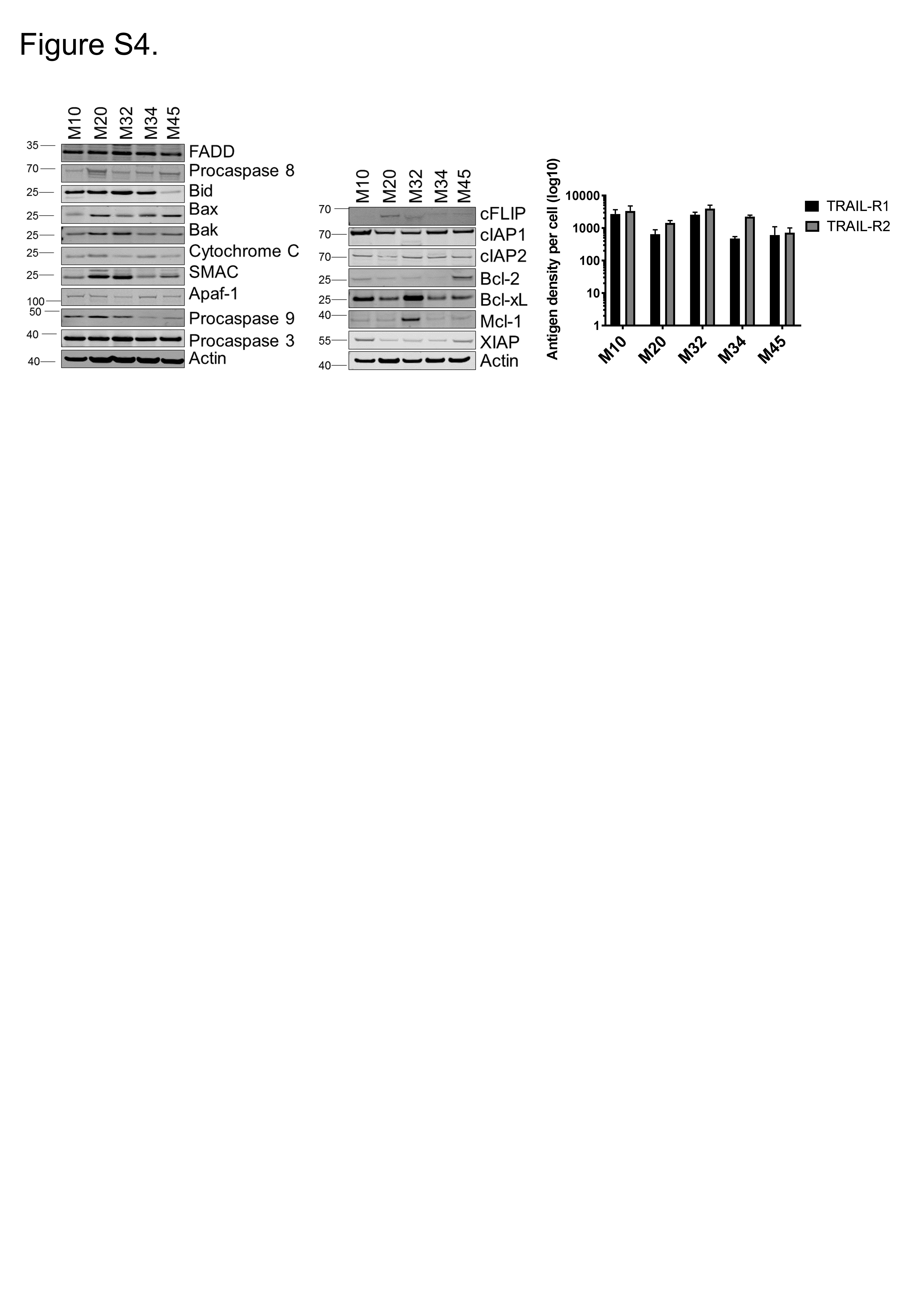

Supplement: Supplementary file 4 — Supplementary figure 4 [file 41418_2020_512_MOESM4_ESM.tif]

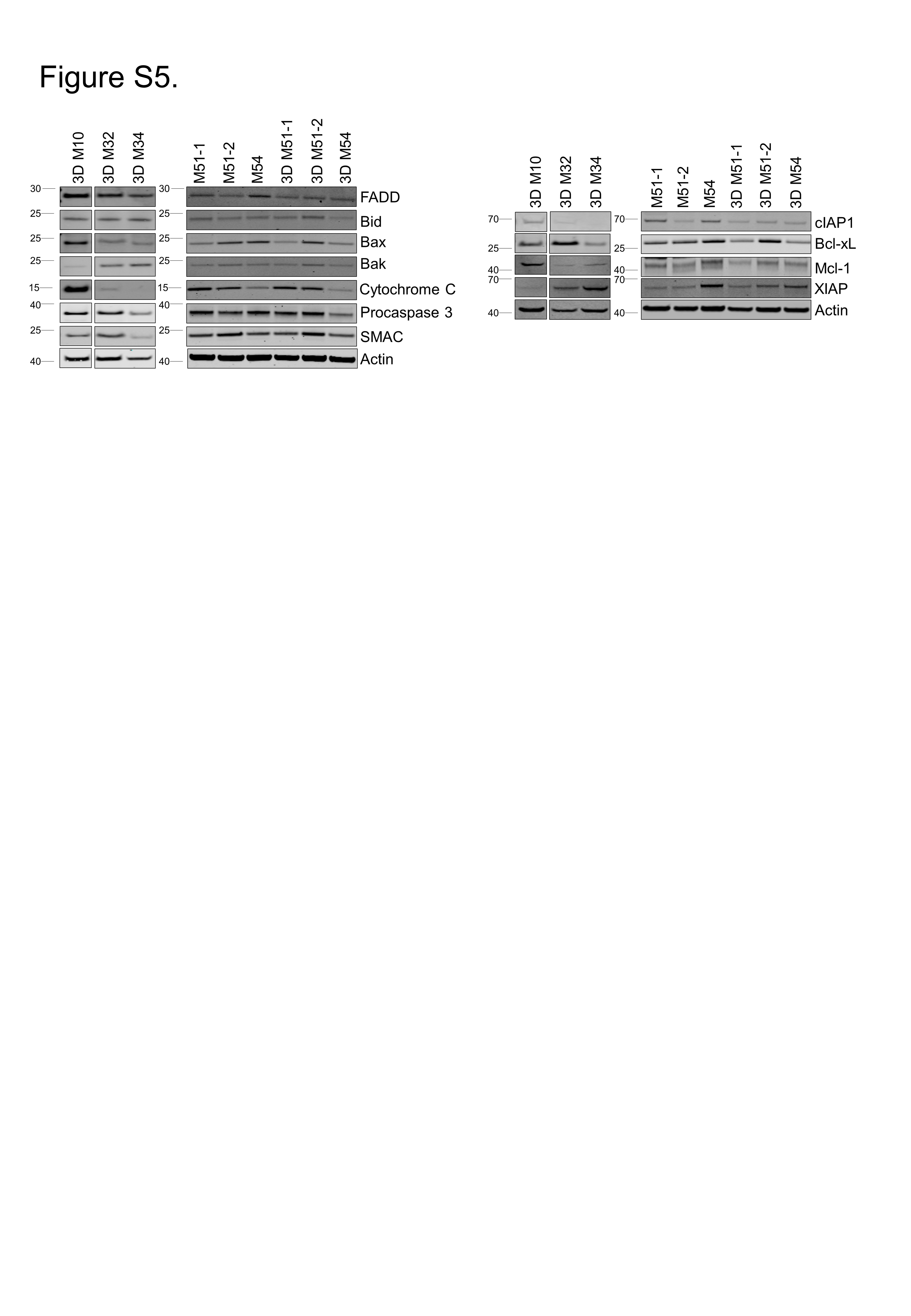

Supplement: Supplementary file 5 — Supplementary figure 5 [file 41418_2020_512_MOESM5_ESM.tif]
