# Supplementary material for: Convergence of pathway analysis and pattern recognition predicts sensitization to latest generation TRAIL therapeutics by IAP antagonism
Source: Cell Death Differ. 2020 Feb 21;27(8):2417–32. doi: 10.1038/s41418-020-0512-5 (PMC7370234; doi:10.1038/s41418-020-0512-5)
Supplement: Supplementary file 8 — Supplementary Figure and Table Legends [file 41418_2020_512_MOESM8_ESM.docx]

**Supplementary Figure Legends**

**Figure S1.** Cell lines MeWo and Mel Juso do not secrete TNF-α when treated with IZI1551, Birinapant or their combination.

A. Titration of recombinant standard TNF-α using Human TNF-α DuoSet ELISA.
B. Cell lines MeWo and Mel Juso do not secrete TNF-α. 3D MeWo and Mel Juso were treated with 1 µM IZI1551, 1 µM Birinapant or their combination for 24 h. Hela cells were exposed to 15 s 30 mJ UVB and treated for 24 h with 10 ng/mL IL-1 for the production of TNF-α that served as a positive control. 100 µl of each supernatant was tested for TNF-α secretion.
C. Histogram of the data presented in B.

**Figure S2.** Apoptosis proteins are heterogeneously expressed in melanoma cell lines. Intracellular pro- and anti-apoptotic proteins were detected by western blotting at high dynamic range. Actin served as loading control. Representative 8 bit converted images are shown from at least n=3 repeat experiments. Death receptors were measured by surface staining and flow cytometry. Bars show mean of antigen density (TRAIL-R1,TRAIL-R2) per cell + SD from n=3 repeat experiments.

**Figure S3.** Protein expression in melanoma MCTS. Cell lines grown as MCTSs were analysed for intracellular pro- and anti-apoptotic protein expression by western blotting. Actin served as loading control. Representative 8 bit converted images are shown from at least n=3 repeat experiments. Death receptors were measured by surface staining and flow cytometry. Bars show mean of antigen density (TRAIL-R1, TRAIL-R2) per cell + SD from n=3 repeat experiments.

**Figure S4.** Protein expression in melanoma cells isolated from patient metastases. Intracellular pro- and anti-apoptotic protein expression was determined by western blotting. Actin served as loading control. Representative 8 bit converted images are shown from at least n=3 repeat experiments. Death receptors were measured by surface staining and flow cytometry. Bars show mean of antigen density (TRAIL-R1, TRAIL-R2) per cell + SD from n=3 repeat experiments.

**Figure S5.** Protein expression in additional melanoma samples, as required for further validation of the reduced predictor. Intracellular pro- and anti-apoptotic protein expression was determined by western blotting. Actin served as loading control. Representative 8 bit converted images are shown.

**Supplemental Table 1**. Protein data obtained from melanoma cell lines and cells isolated from metastases.

**Supplemental Table 2**. Patient demographics and clinical information of the metastatic SKCM-TCGA sub-cohort (n = 365 patients).
